# Neurotrophin-3 accelerates reendothelialization through inducing EPC mobilization and homing

**DOI:** 10.1515/biol-2020-0028

**Published:** 2020-05-11

**Authors:** Yan Chen, Jian Cao, Weixia Peng, Wen Chen

**Affiliations:** Department of Integrated TCM & Western Medicine, Central Hospital of Yiyang city, Yiyang Hunan, 413200, China; Department of General Surgery, Central Hospital of Yiyang city, Yiyang, Hunan, 413200, China; The 8th Medical Center of Chinese PLA General Hospital, Beijing, 100091, China

**Keywords:** neurotrophin-3, endothelialization, endothelial progenitor cells, intravascular stent

## Abstract

Rapid endothelialization is an effective way to treat intimal hyperplasia after intravascular stent implantation. Blood vessels and nerves coordinate with each other in function, while neurotrophin-3 (NT-3) is an important class of nerve growth factors. Our study found that NT-3 promoted endothelial progenitor cell (EPC) mobilization, and the proportion of EPCs in peripheral blood was increased by 1.774 times compared with the control group. Besides, NT-3 promoted the expression of stromal cell-derived factor-1α (SDF-1α), matrix metalloproteinase-9 (MMP9), and chemokine (C-X-C motif) receptor 4 (CXCR4) in EPCs, which increased by 59.89%, 74.46%, and 107.7%, respectively, compared with the control group. Transwell experiments showed that NT-3 enhanced the migration of EPCs by 1.31 times. Flow chamber experiments demonstrated that NT-3 captured more circulating EPCs. As shown by ELISA results, NT-3 can promote the paracrine of vascular endothelial growth factor, interleukin-8, MMP-9, and SDF-1 from EPCs. Such increased angiogenic growth factors further accelerated the closure of endothelial cell scratches. Additionally, EPC-conditioned medium in the NT-3 group significantly inhibited the proliferation of vascular smooth muscle cells. Then animal experiments also illustrated that NT-3 prominently accelerated the endothelialization of injured carotid artery. In short, NT-3 accelerated rapid reendothelialization of injured carotid artery through promoting EPC mobilization and homing.

## 1 Introduction

Vascular stent is the most effective treatment of various cardiovascular diseases, which can significantly improve clinical symptoms of patients [1]. Currently, drug-eluting stents are mainly used in clinics, which effectively prevent intimal hyperplasia and restenosis by inhibiting pathologic proliferation of smooth muscle cells. However, in recent years, more and more clinical data showed that late stent thrombosis and long-term major adverse cardiac events have increased significantly, though drug-eluting stents can reduce early restenosis [2]. This is mainly due to the following: first, while the coating drugs inhibit smooth muscle cell proliferation, they also inhibit the growth of normal cells, thus delaying the self-healing process of blood vessels. Second, drug-coated stents carry a very small amount of drug, and when the drug is released, the stent metal is exposed, stimulating blood vessels and inducing long-term restenosis lesions [3,4]. Therefore, it is of great significance to find new targets for the construction of new-generation intravascular stents.

Endothelial progenitor cell (EPC) is a kind of precursor cell that can be directly differentiated into vascular endothelial cell [5]. EPCs can home to the injured vessels and differentiate into endothelial cells, thereby reducing intimal hyperplasia, thrombosis, and restenosis rate [6]. In addition, EPCs can secrete cytokines such as vascular endothelial growth factor (VEGF), interleukin-8 (IL-8), and stromal cell-derived factor-1 (SDF-1) in a paracrine manner, further promoting the proliferation of endothelial cells [7]. Therefore, the mobilization and homing of EPCs may be an effective way to inhibit restenosis after stent implantation.

Nerves and blood vessels are closely associated with each other in anatomy, and they functionally regulate each other. Neurotrophic molecules can induce the mobilization and homing of EPCs as well as promote endothelialization of tissue-engineering blood vessels. Brain-derived neurotrophic factor (BDNF) can promote the paracrine of vascular growth factor from EPCs and induce angiogenesis [8]. Neurotrophin-3 (NT-3) is an important neurogrowth factor which helps in stimulating and controlling the occurrence of nerves and has been used in the construction of tissue-engineering nerve scaffolds [9]. NT-3 can also be used as a vascular growth factor to treat lower extremity ischemia [10]. Recent study has found that NT-3 promoted angiogenesis and fracture healing by increasing the expression of VEGF [11]. Therefore, we hypothesized that NT-3 can promote rapid endothelialization of injured vessels, thus providing a new target for the construction of new-generation intravascular stents.

## 2 Materials and methods

### 2.1 Isolation and culture of EPCs

Mononuclear cell layer was isolated from mouse heart blood by density gradient centrifugation. EPCs were cultured in the previous method [12]. In short, isolated mononuclear cells were cultured in endothelial growth medium-2 complete medium (Hyclone) containing 10% fetal bovine serum (Hyclone). After 24 h, the unattached cells were removed with phosphate-buffered saline (PBS) and the attached cells continued to be cultured. After culturing for 7 days, the cells were incubated with 5 µl of CD34 (BD Biotec), 10 µl of CD133 (Miltenyi Biotec), and 10 µl of VEGFR-2 (BD Biotec) antibodies at room temperature for 20 min, which was followed by flow cytometry to detect CD34+ CD133+ VEGFR-2+ EPCs.


**Ethical approval:** The research related to animal use has been complied with all the relevant national regulations and institutional policies for the care and use of animals. All animal experiments were approved by the Ethics Committee of Yiyang Central Hospital (Yiyang, China).

### 2.2 EPC mobilization experiment

The experiment was divided into two groups: control group (intraperitoneal injection of the same dose of physiological saline) and NT-3 group (intraperitoneal injection of 15 mg/kg NT-3). After 7 days of continuous intraperitoneal injection, heart blood of C57BL/6 mice was collected and mixed with equal volume of PBS; transferred to percoll separation solution (Sigma) with 1.5 times volume; centrifuged for 30 min; resuspended in 50 μl of medium; and stained with CD34, VEGFR-2, SDF-1α, matrix metalloproteinase-9 (MMP9), or chemokine (C-X-C motif) receptor 4 (CXCR4) antibody. Cells were washed with PBS for three times and then detected by flow cytometry.

### 2.3 EPC migration experiment

EPCs were divided into the control group and NT-3 group. VEGF or NT-3 was added into the lower chamber, and 1 × 10^4^ EPCs were added to the upper layer of the transwell chamber. After incubating for 12 h, the cells in the upper layer were removed, fixed with 4% polyformaldehyde, and stained with 0.5% crystal violet (Sigma). Then the number of positive cells was calculated under a microscope.

### 2.4 Flow chamber experiment

EPCs were divided into the control group and NT-3 group. The glass slides coated with VEGF or NT-3 were placed in the flow chamber and then EPCs were made into cell suspensions of 1 × 10^5^/ml cell density and flowed through the slides at a rate of 0.01 ml/min. EPCs attached to the glass slides were detected by an inverted microscope.

### 2.5 Detection of cytokines

EPCs were divided into the control group and NT-3 group. After stimulating for 48 h, the supernatant was discarded. EPCs were washed with PBS for three times, and fresh culture was added. After continuous culture for 48 h, the supernatant was collected, and VEGF, IL-8, MMP-9, and SDF-1 concentrations in the supernatant were detected by ELISA (RD).

### 2.6 Coculture experiment

Vascular smooth muscle cells were cultured in DMEM/F12 plus 10% fetal bovine serum. When smooth muscle cells were in the logarithmic growth phase, they were digested with 0.25% trypsin (Hyclone) and passed to the 24-well plate (Corning) and then EPC-conditioned medium (CM) was added. The cells were divided into three groups: control CM group, EPC CM group (1 : 1 ratio added to EPC supernatant of control group), and NT-3 CM group (1 : 1 ratio added to EPC supernatant of NT-3 group). After 24 h culture, 5-ethynyl-2-deoxyuridine (EDU) dyeing reaction fluid (Ribo) was added. After continuous culture for 6 h, the culture medium was discarded and the cells were washed 1–2 times for 5 min each time. Each hole was fixed with 4% polyformaldehyde for 30 min and then incubated with 2 mg/ml glycine for 5 min. After three times of PBS cleaning, PBS with 0.5% TritonX-100 (Sigma) was added and then the mixture was incubated for 10 min. To each group EDU dyeing solution (Ribo) was added, avoiding light, and incubated with decolorization of bed for 30 min at room temperature. Cleaning with 0.5% TritonX-100 PBS for three times was followed by 4′,6-diamidino-2-phenylindole dyeing (Sigma) and mounting. Then the cells were observed under a fluorescence microscope.

### 2.7 Animal experiment

Eight-week-old mice were selected and then anesthetized with 0.3% pentobarbital sodium. The external carotid artery was ligated, and the internal carotid artery and common carotid artery were clipped temporarily at the same time. The guide wire was inserted into the common carotid artery through the external carotid artery and moved back and forth for ten times. After establishing common carotid artery wire injury, NT-3 (15 mg/kg) or VEGF (10 nm/kg) injection was administered intraperitoneally. Forty-eight hours after administering the injection, the injured segment of the common carotid artery was obtained and fixed by 4% paraformaldehyde. Then paraffin sections were taken and immunohistochemical staining of CD31 was performed to observe endothelialization.

### 2.8 Statistical method

All the experimental data were expressed as mean ± SD. Variance analysis and *t* test were performed by using SPSS 17.0 software. *P* < 0.05 was considered a statistically significant difference.

## 3 Results

### 3.1 Identification of EPCs

**Figure 1:**
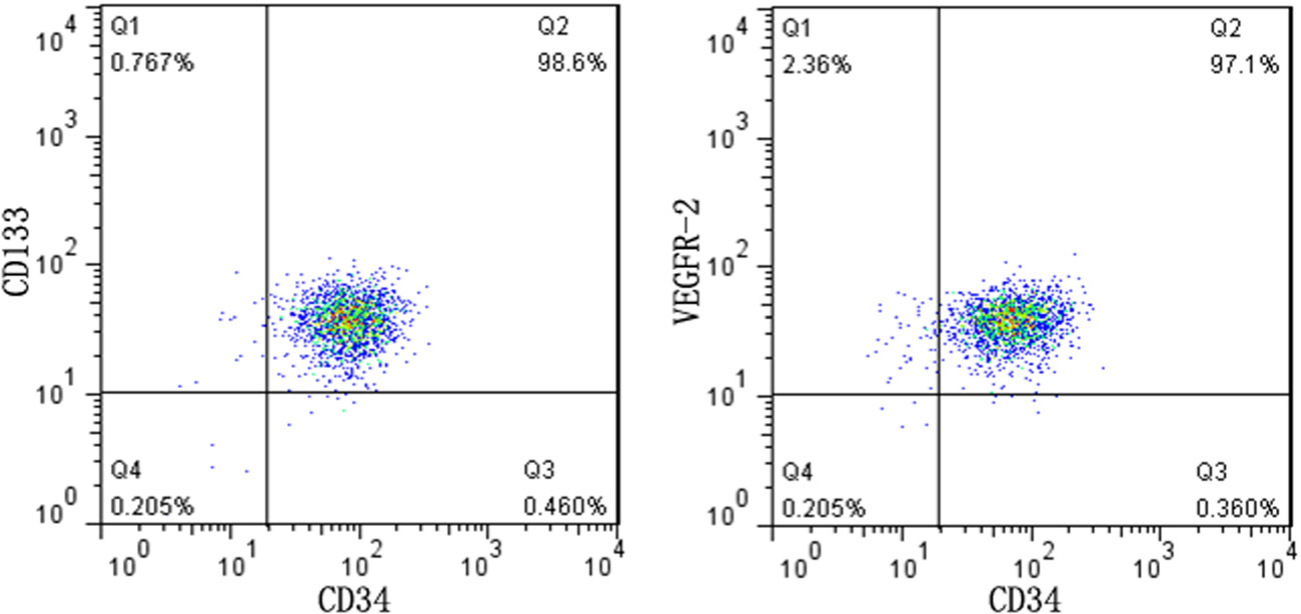
Identification of EPCs. Over 90% of cultured cells expressed CD34, CD133, and VEGFR-2.

Flow cytometry results showed that after 7 days of culture, more than 90% of cells expressed CD34, CD133, and VEGFR-2 at the same time, indicating that our cultured cells were EPCs (Figure 1).

### 3.2 NT-3 promoted the mobilization of EPCs

**Figure 2:**
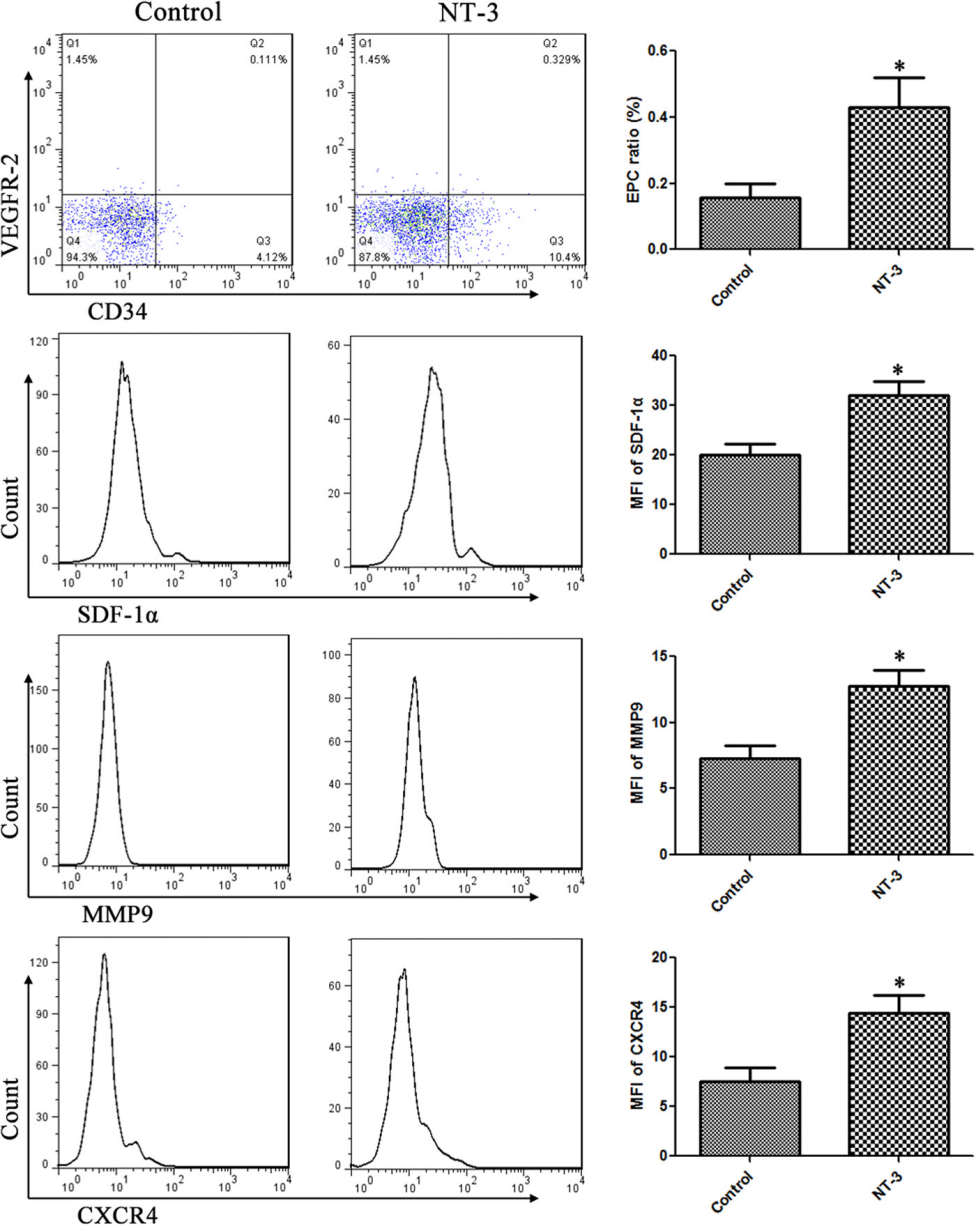
NT-3 promoted EPC mobilization. The proportion of EPCs in the NT-3 group was 1.774 times higher than that in the control group. NT-3 promoted the expression of SDF-1α, MMP9, and CXCR4 in EPCs, which increased by 59.89%, 74.46%, and 107.7%, respectively, compared with those of the control group. *P < 0.05 (*n* = 10) versus control. Values are expressed as mean ± SD.

In order to detect EPC mobilization, we calculated EPC proportion in peripheral blood. The results showed that NT-3 could significantly promote the increase of EPCs in peripheral blood, which increased by 1.774 times compared with that of the control group (*t* = 2.747, *P* = 0.0206). We further studied the mechanism, and the results showed that NT-3 promoted the expression of SDF-1α, MMP9, and CXCR4 in EPCs, which increased by 59.89%, 74.46%, and 107.7%, respectively, compared with the control group (*t* = 3.284, *P* = 0.0082; *t* = 3.490, *P* = 0.0058; and *t* = 4.337, *P* = 0.0015) (Figure 2).

### 3.3 NT-3 promoted EPC migration

**Figure 3:**
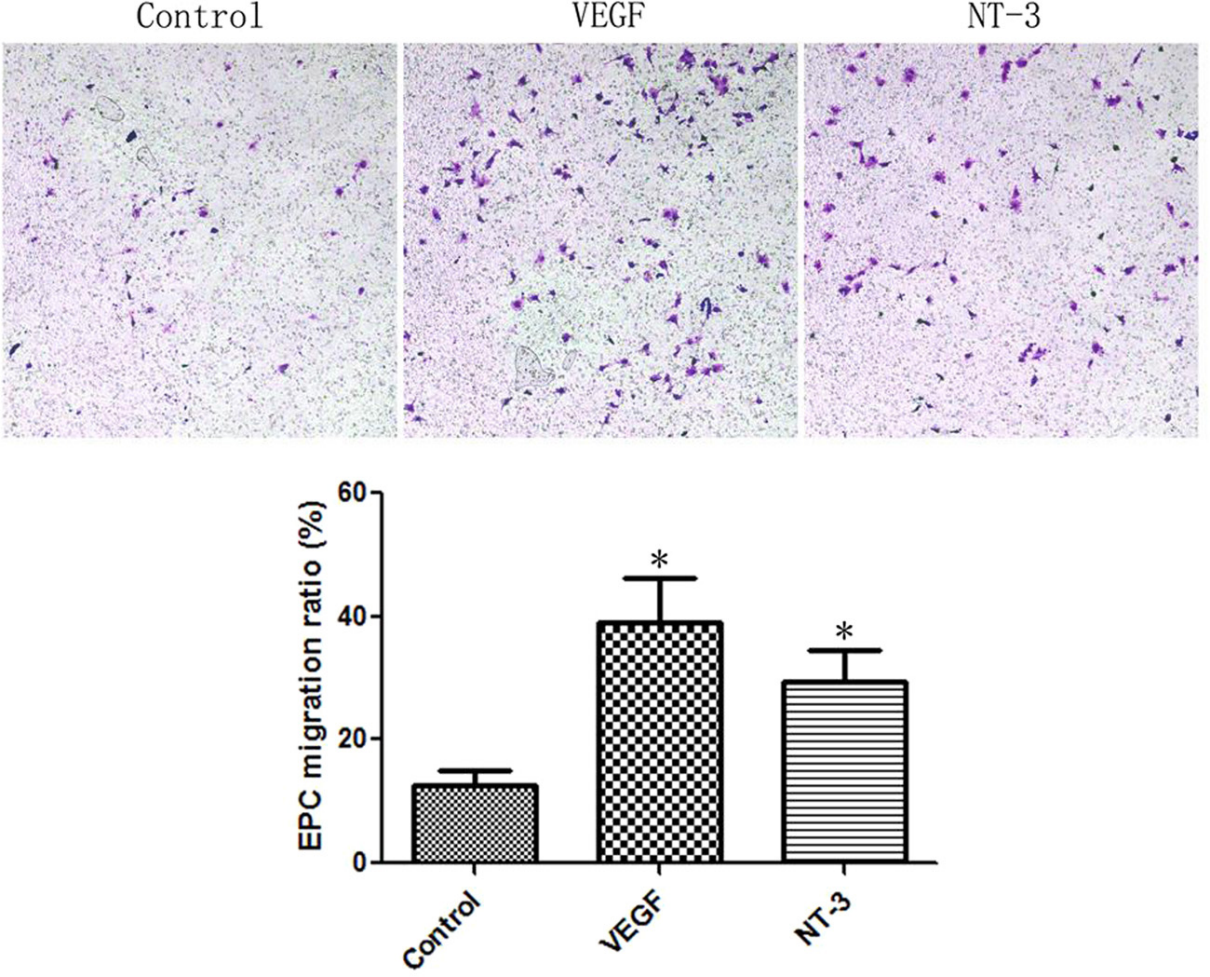
NT-3 promoted EPC migration. The proportion of migrated cells in the NT-3 group was 1.31 times higher than that of the control group. *P < 0.05 (*n* = 6) versus control. Values are expressed as mean ± SD.

In order to detect the effect of NT-3 on EPC migration, we used the transwell experiment. The results showed that VEGF could significantly promote the migration of EPCs, which increased by 2.07 times compared with the control group (*t* = 3.389, *P* = 0.0276). NT-3 also significantly promoted the migration of EPCs, which was 1.31 times higher than that of the control group (*t* = 2.881, *P* = 0.0450) (Figure 3).

### 3.4 NT-3 effectively captured circulating EPCs

**Figure 4:**
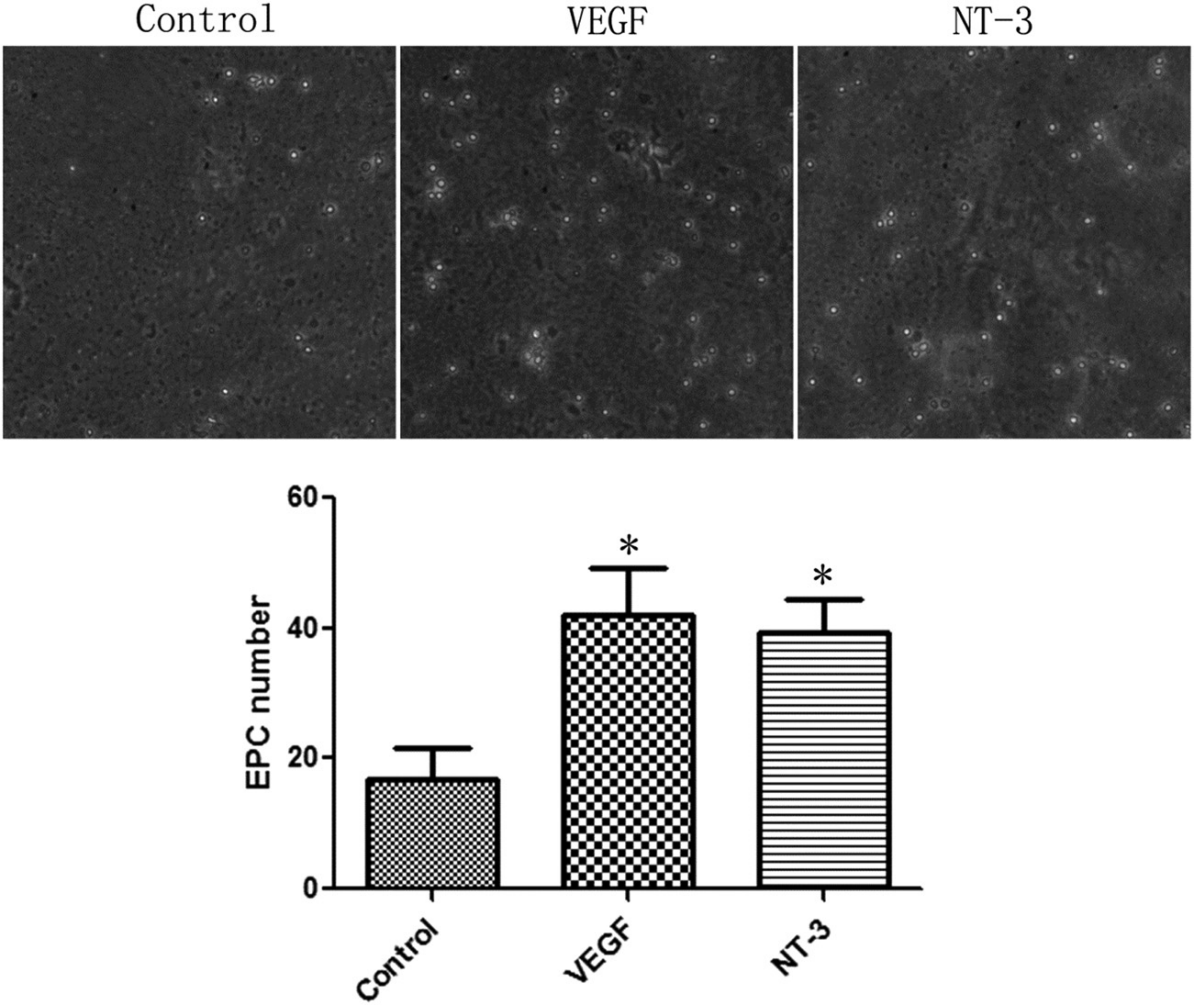
NT-3 effectively captured circulating EPCs. Compared with the control group, the number of captured cells in the NT-3 group increased by 1.33 times. *P < 0.05 (*n* = 6) versus control. Values are expressed as mean ± SD.

The results of flow chamber experiments showed that both VEGF and NT-3 could significantly capture more circulating EPCs. Compared with the control group, the number of captured cells in VEGF and NT-3 groups increased by 1.49 and 1.33 times, respectively (*t* = 2.869, *P* = 0.0455; *t* = 3.182, *P* = 0.0335) (Figure 4).

### 3.5 NT-3 promoted the paracrine of EPCs

**Figure 5:**
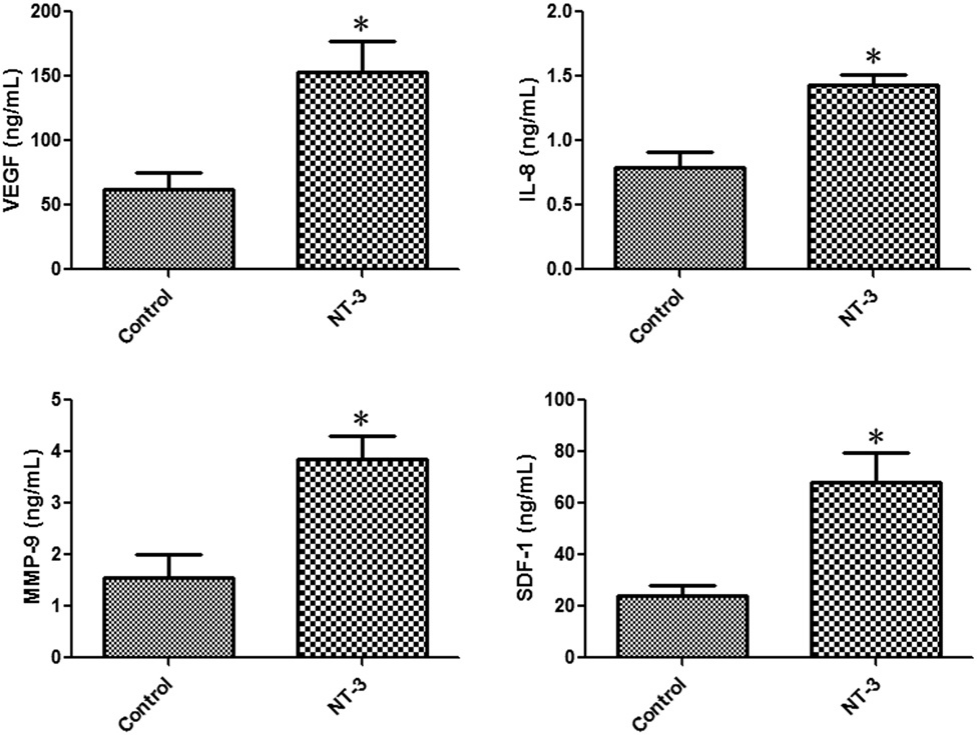
NT-3 promoted the paracrine of EPCs. The contents of VEGF, IL-8, MMP-9, and SDF-1 in the NT-3 group increased by 1.46 times, 81.39%, 1.47 times, and 1.82 times, respectively. *P < 0.05 (*n* = 6) versus control. Values are expressed as mean ± SD.

**Figure 6:**
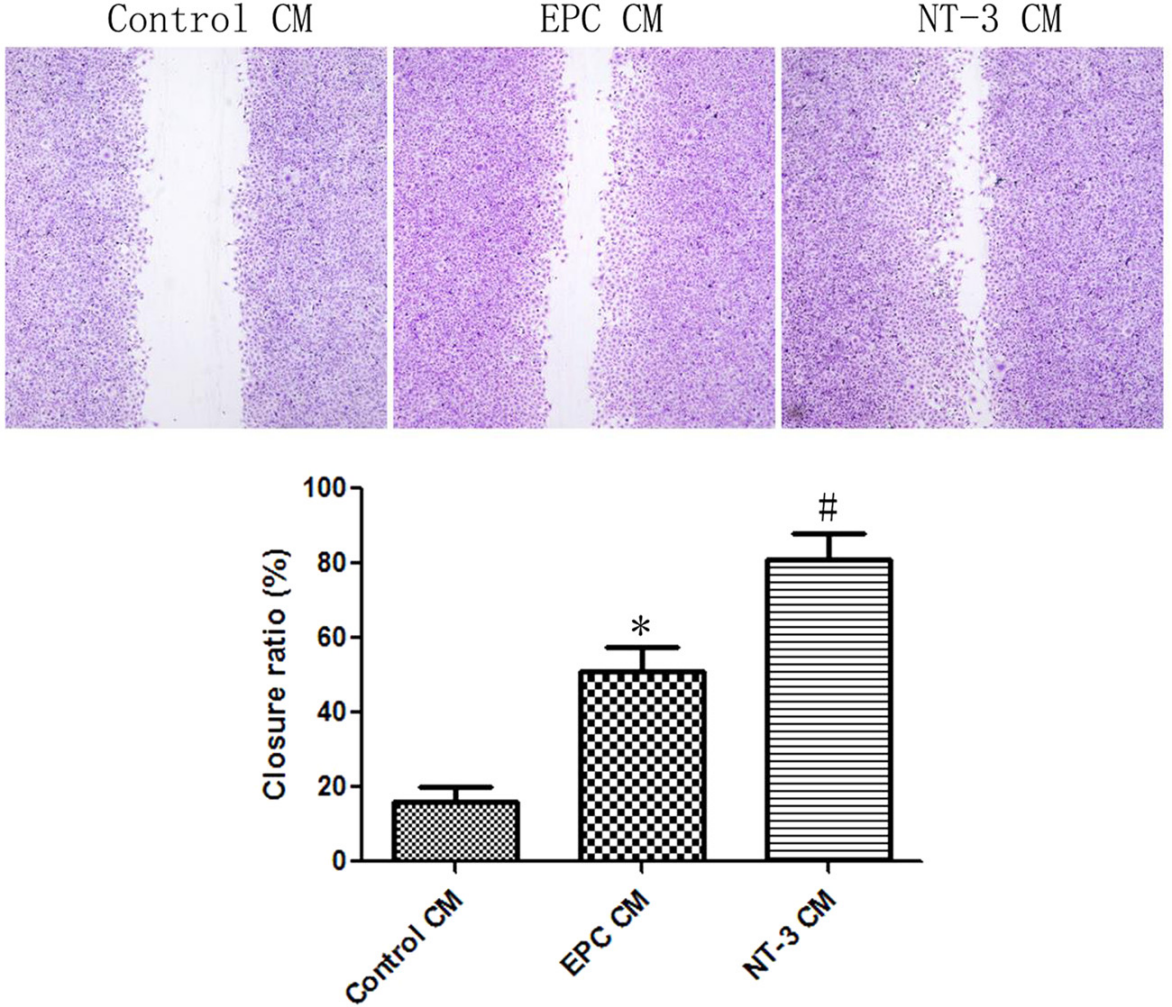
EPC CM in the NT-3 group promoted scratch closure of endothelial cells. *P < 0.05 (*n* = 6) versus control CM; ^#^ *P* < 0.05 (*n* = 6) versus EPC CM. Values are expressed as mean ± SD.

The ELISA results showed that compared with the control group, VEGF, IL-8, MMP-9, and SDF-1 concentrations in the supernatant of the NT-3 group increased by 1.46 times (*t* = 3.269, *P* = 0.0308), 81.39% (*t* = 4.313, *P* = 0.0125), 1.47 times (*t* = 3.753, *P* = 0.0199), and 1.82 times (*t* = 3.673, *P* = 0.0213), respectively (Figure 5). These increased angiogenic factors further promoted scratch closure of endothelial cells (Figure 6).

### 3.6 EPC CM in the NT-3 group inhibited the proliferation of smooth muscle cells

**Figure 7:**
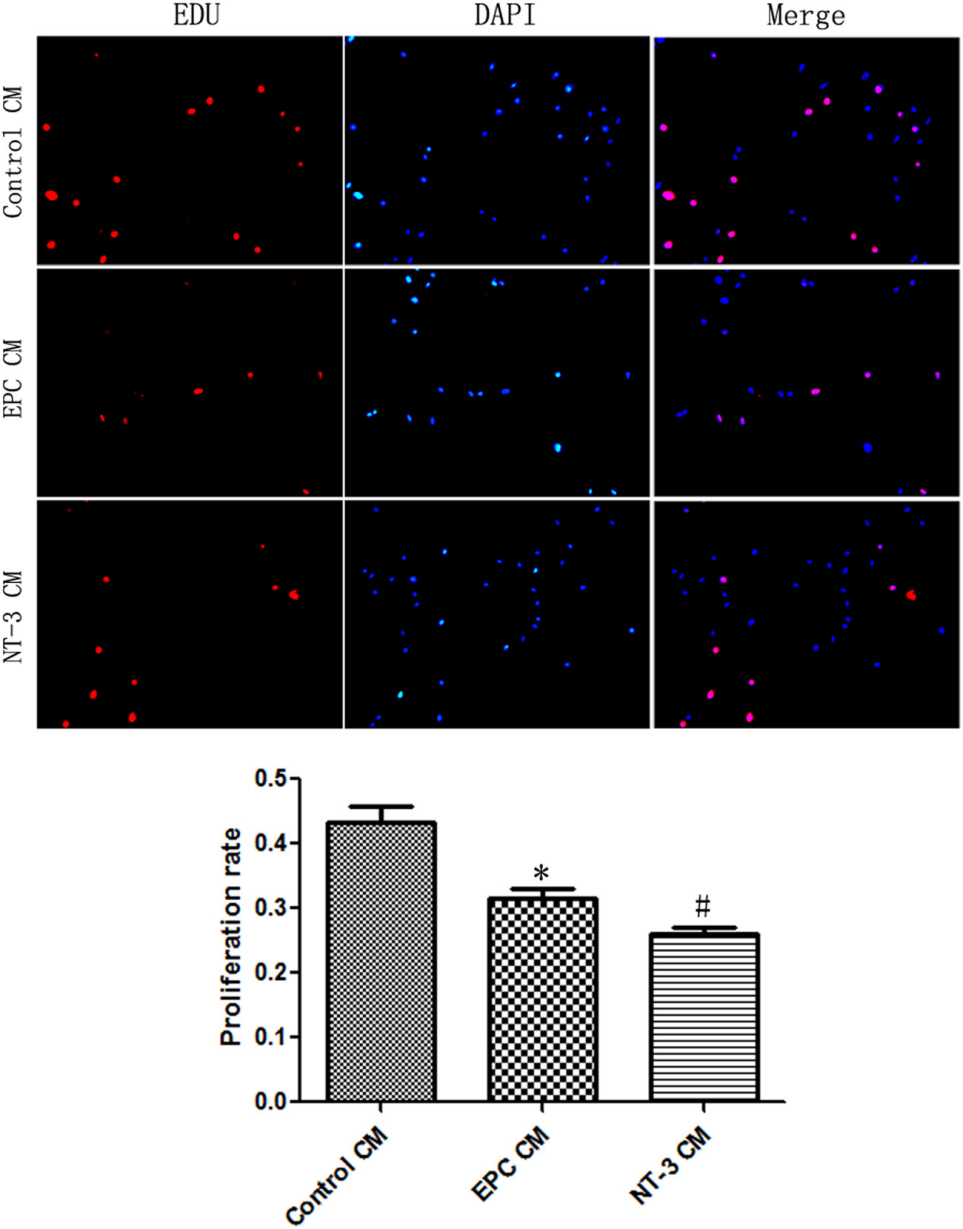
EPC CM in NT-3 group inhibited the proliferation of smooth muscle cells. *P < 0.05 (*n* = 6) versus control CM; ^#^ *P* < 0.05 (*n* = 6) versus EPC CM. Values are expressed as mean ± SD.

Pathological proliferation of smooth muscle cells is closely related to intimal hyperplasia after stent implantation. Many studies have reported that the paracrine of EPC can inhibit the proliferation of smooth muscle cells [13,14]. We used EDU to detect the proliferation of vascular smooth muscle cells. Compared with the control CM group, the EPC CM inhibited the proliferation of vascular smooth muscle cells. NT-3 CM further inhibited the proliferation of smooth muscle cells, which was reduced by 17.91% compared with the EPC CM group (*t* = 3.053, *P* = 0.0379) (Figure 7). These results indicated that NT-3 can significantly inhibit the proliferation of smooth muscle cells by enhancing the paracrine of EPCs.

### 3.7 NT-3 promoted rapid endothelialization of injured arteries

**Figure 8:**
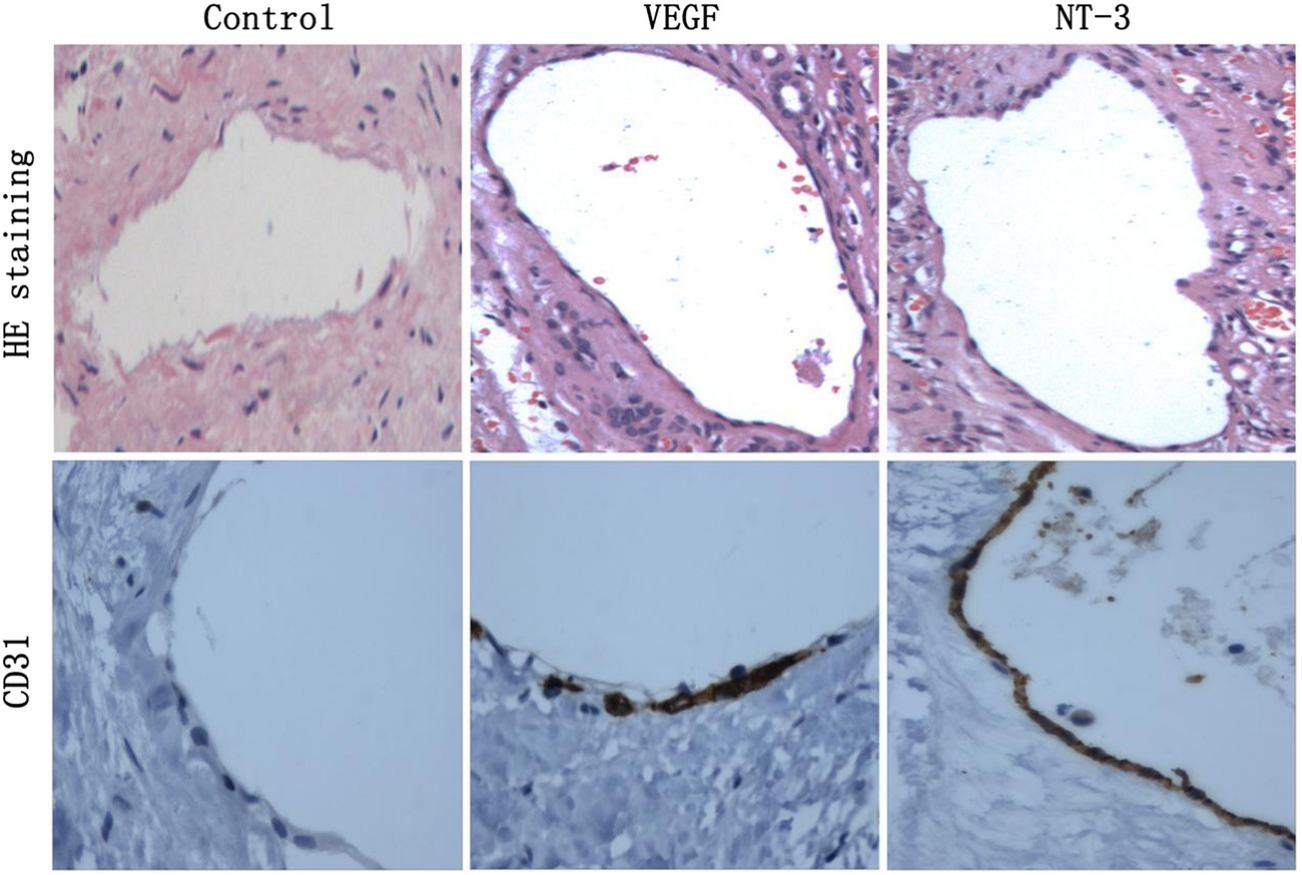
NT-3 promoted rapid endothelialization of injured arteries. HE staining showed that both VEGF and NT-3 could significantly promote rapid endothelialization of injured carotid arteries. Immunohistochemistry showed that injured carotid arteries in the NT-3 group formed a complete endothelial cell layer.

The results of HE staining showed that both VEGF and NT-3 significantly promoted rapid endothelialization of injured carotid arteries. Immunohistochemistry showed that injured carotid arteries in the NT-3 group formed a complete endothelial cell layer (Figure 8).

## 4 Discussion

Drug-eluting stents are the main stent for the interventional treatment of cardiovascular and cerebrovascular diseases, which can significantly improve patients’ symptoms, whereas their long-term effects are not ideal. Intimal hyperplasia is the main cause of stent implantation failure. Endothelial cells have the important functions in inhibiting thrombosis and intimal hyperplasia, due to which promoting rapid endothelialization can significantly inhibit intimal hyperplasia [15]. EPC has many specific surface markers, such as VEGFR-2, CD34, and CD133 [16]. Scientists constructed CD34 antibody-coated stents for EPC capture. The clinical results showed that this stent could rapidly capture EPCs, so that the stent formed a single endothelial cell layer within 2 days. Rapid endothelialization could effectively inhibit the formation of thrombosis and restenosis [17,18,19]. However, CD34+ cells in peripheral blood can differentiate not only into endothelial cells but also into smooth muscle cells. It has been found that anti-CD34 antibody-modified grafts promoted rapid endothelialization but stimulated intimal hyperplasia in arteriovenous expanded polytetrafluoroethylene grafts [20]. Our results suggested that NT-3 promoted the mobilization and homing of EPCs. Besides, NT-3 can significantly inhibit the proliferation of smooth muscle cells by enhancing EPC paracrine. So NT-3 can be used as an effective target for the construction of EPC-captured intravascular stents.

The nerves and vessels regulate each other in function. Blood vessels can provide the nutrients and oxygen needed for nerve regeneration; in addition, vascular growth factor can directly promote the ingrowth of nerves [21,22]. Although drug-eluting stents can promote endothelialization, they did not improve the immune microenvironment and promote the repair of nerve injury. The study found that many nerve molecules were proangiogenic factors. BDNF can significantly promote the mobilization and homing of EPCs and then induce rapid endothelialization of tissue-engineering blood vessels [8]. Netrin-1 possesses a strong function of promoting angiogenesis and is an effective treatment of lower limb ischemia [23,24]. NT-3 is an important neurotrophic factor that can induce the ingrowth of nerve endings, and the nerve reconstruction can effectively maintain the stable internal environment of vascular tissues [25]. This study found that NT-3 can also induce EPC mobilization, homing, and promote paracrine, thereby promoting rapid endothelialization of injured blood vessels. Rapid endothelialization plays an important role in the long-term inhibition of intimal hyperplasia. When a stable system is formed locally, the stent will maintain a lasting patency.

EPCs are essential for repairing arterial injury [26]. Our results suggested that NT-3 can promote EPC mobilization into the peripheral blood. Increased EPCs in the peripheral blood can further home to the injured part under NT-3. In contrast, EPCs can directly differentiate into endothelial cell island, promoting the proliferation and migration of endothelial cells around the injured side by paracrine. Through these two mechanisms, NT-3 promoted rapid endothelialization of injured arteries and ultimately achieved the aim of inhibiting intimal hyperplasia.

In conclusion, our study found that NT-3 induced rapid endothelialization of injured carotid arteries by inducing EPC mobilization and homing. It provided a new target for the construction of EPC-capture intravascular stents.

## Figures and Tables

**Figure A1:**
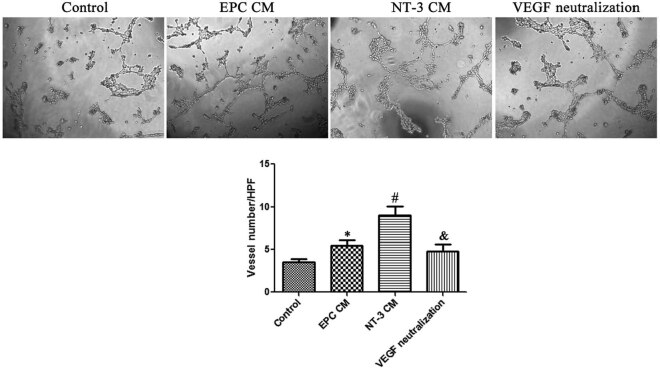
NT-3 regulated the ability of EPCs to generate vessels. Compared with the EPC CM group, the number of vessels in the NT-3 CM group significantly increased. VEGF neutralization significantly inhibited the effect of NT-3.
